# Spectroscopic Evidence of Thermal Changes in Plant Oils during Deep-Frying—Chemical and Infrared Studies

**DOI:** 10.3390/plants11141813

**Published:** 2022-07-09

**Authors:** Lucyna Dymińska, Abduladhim Moamer Moftah Albegar, Wojciech Sąsiadek, Edyta Kucharska, Adam Zając, Jerzy Hanuza

**Affiliations:** 1 Department of Bioorganic Chemistry, Wroclaw University of Economics and Business, Komandorska 118/120, 53-345 Wrocław, Poland; lucyna.dyminska@ue.wroc.pl (L.D.); wojciech.sasiadek@ue.wroc.pl (W.S.); adam.zajac@ue.wroc.pl (A.Z.); 2 Faculty of Agriculture, Azzaytuna University, Bani Walid, Libya; aalbegar@yahoo.com; 3 Institute of Low Temperature and Structure Research, Polish Academy of Sciences, Okólna 2, 50-422 Wrocław, Poland; j.hanuza@int.pan.wroc.pl

**Keywords:** thermal degradation, FT-IR spectra, plant oil, deep-frying effect, fatty acids, carotenoids, antioxidants

## Abstract

For this study, the thermal degradation of palm, coconut, rice bran, and rapeseed (canola) oils was studied. Products formed during deep-frying were identified using chemical methods and these results were verified with those derived from FT-IR (Fourier-transform infrared spectroscopy) studies. Mathematically processed spectral data were analyzed in terms of the breaking of double bonds, the decomposition of the carotenoids, and the reduction of the C=O carbonyl group. Clearly visible changes in the position and intensity of some bands were used for explaining the structural changes in the studied oils. These changes prove that during the heating of the oils, decomposition of the plant fat into fatty acids appears, together with the reduction of the number of certain bonds (e.g., C=C, =C-H, and C=O) and cracking of the acylglycerol chains. The iodine values of the heated oils, determined from the FT-IR spectra measurements, show a significant decrease in their degree of unsaturation level. These effects, visible in the FT-IR spectra, confirm the chemical and structural changes derived from the chemical and physicochemical studies of the plant oils. The influence of heating time on the band intensity of proteins was also studied.

## 1. Introduction

The deep-frying of food products in oils is one of the most popular processes in their preparation for consumption. Fried food gains a specific flavor, color, and texture [1]. During the frying, the food has contact with the hot oil, air, and the internal fat of the fried product. Frying produces several flavor compounds and leads to chemical changes in the food and oil [2]. There are some processes, not only hydrolysis and oxidation but also polymerization, that cause degradation of the food and oil, especially tocopherols and amino and fatty acids. It was reported that these reactions depend on several factors, namely: oil and food quality, the type of fryer, frying conditions (including access to oxygen), frying temperature, and the frequency of replenishment with fresh oil [2]. The oxidative stability of the oil used strongly depends on the temperature of frying, the volume of fried food (the number and mass), the content of free and unsaturated fatty acids, the presence of polyvalent metal ions, and the number of antioxidants in the food and oil [2]. It was also shown that intermittent frying causes greater quality degradation in the oil than continuous frying [3]. Since 2001, numerous works have been dedicated to the thermal stability of different kinds of oils, and the results of their degradation were studied with the use of various methods.

Corn and soybean oils were investigated by differential scanning calorimetry to establish the microwave heating effects on their cooling profiles and to compare them with changes in the chemical parameters [4]. It was reported that the power of microwave heating decides the unsaturated triacylglycerol (TAG) disappearance level and the formation of more saturated TAG. In the past, similar studies with the use of microwave heating were performed [5,6,7].

The thermal diffusivity of commercially available edible oils was determined via thermal lens spectroscopy, in which a 632.8 nm He-Ne laser line and 488 nm Ar laser beam were applied as the probe and excitation, respectively. Canola, peanut, olive, sunflower, maize, and soybean oils were studied. It was reported that the UV/Vis spectra exhibit a significant shift that is dependent on the relative concentration of saturated and unsaturated carboxylic acids [8].

The thermal behavior of rice bran, sunflower oils, and their mixtures was examined to determine changes in their physicochemical parameters, such as acid value, iodine value, color value, peroxide value, and fatty acids. Repeated deep-fat-frying processes were performed using dried potato chips, showing good thermal stability of the studied oils and their blends; model blends consisting of 60% rice bran oil and 40% sunflower oil were particularly effective [9]. Similar studies were conducted on mixed and inter-esterified blends of coconut or palm oil with rice or sesame bran oil. The composition and thermal profiles of the mixtures were determined by differential scanning calorimetry and the HPLC method [10]. Other oils were selected for the investigation of heat-induced physicochemical changes at frying temperatures. The chemical (free fatty acid content, peroxide value, iodine value) and physical (viscosity, refractive index, color) properties of hazelnut, olive pomace, grapeseed and sunflower oils were identified. The best thermal performances were found for hazelnut and olive pomace oils [11]. It was also found that hazelnut oil was more stable than corn and sunflower oils [12].

The thermal stability of selected active principles of extra virgin olive oils (mainly phenolic compounds) was studied by isotope dilution liquid chromatography and tandem mass spectrometry. The results led to the conclusion that applying a conventional heating process makes the oils undergo obvious degradation [13].

The stability of essential oils stored under various conditions was extensively investigated, and the results were reviewed [14]. The various paths of degradation upon exposure to extrinsic parameters were outlined; the temperature, light, and oxygen atmosphere were particularly recognized as having a crucial impact on essential oil integrity. Oxidative changes in sunflower, grape seed, soybean, corn, and olive oils caused by frying temperature were studied using chromatographic and chemical methods. It was reported that olive oil has better stability against thermal oxidation when compared to the stability of polyunsaturated oils. On the other hand, among the unsaturated oils, corn and soybean oils are the most resistant to degradation at frying temperatures [15].

Lately, some new information on the thermal stability of a wide class of edible oils has been published. In the majority of these reports, the results of chemical studies were the basis for analysis of the effects occurring at high temperatures. The blending mixtures of palm and sesame oils with different ratios were investigated to achieve blends with a low cost and better nutritional advantages [16]. The influence of heat and oxidative changes with temperature on soybean oil was considered, in terms of its harmful effects and health risks regarding the consumption of food fried in degraded oil [17]. It was reported that the addition of antioxidants improves the resistance of vegetable oils to thermal degradation processes during frying. Such investigations were focused on canola and vegetable oil, as well as sesame, sunflower, and palm oils [18,19]. The effect of thermal oxidation was studied using the example of arachis oil, which has a high smoke point and an intense color originating from a high content of β-carotene and lutein [20]. Ultra-fast gas chromatography coupled with chemometrics was applied in studies of the thermal degradation of canola and olive oils. It was demonstrated that the addition of olive oil improves the stability of rapeseed oil and changes its flavor and aroma profile [21].

Applying spectroscopic methods in the investigation of the thermal properties of vegetable oils is not common. The heat-induced degradation of carotenoids in extra virgin olive oil was studied during both microwave and conventional heating processes [22]. A progressive degradation in carotenoids was observed, starting at 180 °C and 140 °C in the microwave and conventional heating methods, respectively. The Raman bands originating from carotenoid content completely disappeared at 203 °C during conventional heating, while these lines were still observed even up to 225 °C during microwave heating. A loss of cis C=C bonds and a decrease in free fatty acid content were also observed in the spectra [22]. In the past, changes in the Raman [23,24] and IR [23,25,26,27] spectra of heated oils were investigated. Sunflower, canola, and olive oils were compared by the authors of [24]; the most obvious degradation was reported for polyunsaturated sunflower oils, but the prolonged heating of cooking oils led to the formation of degradation products affecting human health.

Some oils are particularly stable during the deep-frying process, e.g., palm, coconut, and rice bran oils. For the preparation of oil mixtures forming a model blend with sunflower oil, rice bran oil was used [9]. The addition of certain oils to other oils (e.g., olive oil to soybean or canola oils) improves the stability of these blends [21]. In our previous work, we concluded, on the basis of our experimental determination of the fatty acid profiles and iodine values, that palm, coconut, rice bran, and rapeseed (canola) oils and the model blends (formed from these oils) can be expected to be relatively resistant to degradation during prolonged frying [28]. They were also chosen due to their low cost and popularity in Poland. In this paper, Fourier transform IR spectroscopy was applied to detect the degradation effects in these oils. The aim of this work was to test if IR was a good tool for detecting the degradation of oils during heating.

## 2. Results and Discussion

### 2.1. Chemical Properties

The chemical characterization of the four oils reported here was presented in our previous paper [28,29]. On the basis of these data, the following comparison of their proposed percentage content of the SFA (saturated fatty acid), MUFA (monounsaturated fatty acid), and PUFA (polyunsaturated fatty acid) fractions in these oils is shown in Table 1.

The highest PUFAs appear in rice bran oil—37.84%, of these, the content of linoleic acid (C18:2n6c) is the highest. Linolenic acid (C18:3n3) is present in a small amount (0.30%). Rapeseed oil also contains a high level of PUFAs—28.07%. This fraction contains linoleic acid (18.84%), α-linolenic acid (8.94%), and a low level of DHA—docosahexaenoic acid (C22:6n3). In palm oil, the PUFA fraction appears only at about 9%. This mainly comprises linoleic acid (8.49%) and small amounts of α-linolenic acid, docosahexaenoic acid, and docosadienoic acid (C22:2). One PUFA—linoleic acid—is found in very small amounts in coconut oil (0.65%). The PUFA content in coconut oil is very small—0.85%.

A similar comparison of the chemical composition can be made for the MUFA fraction. Rapeseed oil contains about 63% of MUFAs. In rice bran oil and palm oil, the level of MUFA is comparable—about 45%. This is mainly from oleic acid (C18:1n9c). This fraction contains palmitoleic (C16:1), eicosenoic (C20:1n9), and erucic (C22:1n9) acids, which appear in small amounts. The MUFA fraction in coconut oil appears only at about 4.5%; it contains elaidic, oleic, and eicosenoic acids.

All the analyzed oils contain the following SFAs: butyric (C4:0), lauric (C12:0), myristic (C14:0), palmitic (C16:0), stearic (C18:0), arachidic (C20:0), behenic (C22:0), and lignoceric (C24:0) acids. Coconut oil contains the highest level of SFA—64.6%. In addition to those saturated fatty acids mentioned above, coconut oil also contains caproic (C6:0), caprylic (C8:0), and capric (C10:0) acids. The lauric and myristic acid contents are the highest in this oil. The palm oil contains 46.10% of the SFA fraction, which mainly comprises palmitic acid. The remaining oils contain small amounts of saturated oils: 16.62% in rice bran oil and 8.91% in rapeseed oil.

A similar comparison can be made when analyzing the mean length of the carbon chains in the studied oils. Taking into account the partial percentage content of particular chains, consisting of 4 to 24 carbon atoms, the following mean chain length can be calculated: 17.74 for rapeseed, 17.51 for rice bran, 17.00 for palm oil, and 12.50 for coconut oil. This sequence should be reflected in the results of the spectroscopic studies.

### 2.2. Heating–Time Dependence of the Iodine Values

The content of SFAs, MUFAs, and PUFAs shall be taken into account in the blending oils intended for deep frying. Their ratios influence the stability of the oils at high temperatures because the saturated fats are more thermally stable and MUFAs are less susceptible to heat and oxidation in multiple heating than PUFAs. Their fatty acid profiles, iodine values, and smoke points differentiate the studied oils and change during the heating process. This test was carried out by frying the oils at 180 °C for 4 h, repeated in 10 cycles, with 24 h intermission between the cycles.

**Figure 1:**
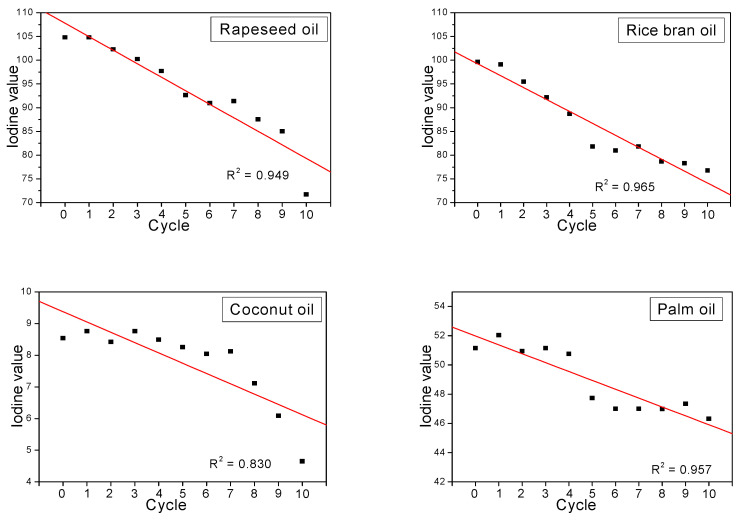
Dependence of the iodine values on heating time. The regression coefficients are from −2.89 (rapeseed oil) to −0.32 (coconut oil).

The dependences of the iodine values presented in Figure 1 show that the prolonged high-temperature treatment of the studied oils influences the structure of the tested oils.

The iodine values of the heated oils determined from the IR spectra measurements show a significant decrease in their degree of unsaturation level. Note that their values decrease from 9 to 4.5 in the case of coconut oil samples, from 52 to 46 for palm oil, from 100 to 77.5 for rice bran oil samples, and from 105 to 72.5 for rapeseed oil. These results agree with their chemical composition, in which the level of the MUFA + PUFA fraction decreases in series: 7–8% for coconut oil, 45–48% for palm oil, 71–81% for rice bran oil, and 88–94% for rapeseed oil. All the studied oils show a quasi-linear relationship, which is clearly seen in the first five heating cycles of the rapeseed and rice bran oils. For the coconut and palm oils, these dependencies not only change irregularly but also show a linear tendency. It should be noted that the correlation coefficient for these relationships was 0.830–0.965, showing a quasi-linear relationship between boiling time and iodine number.

### 2.3. Infrared Spectra Measurements

**Figure 2:**
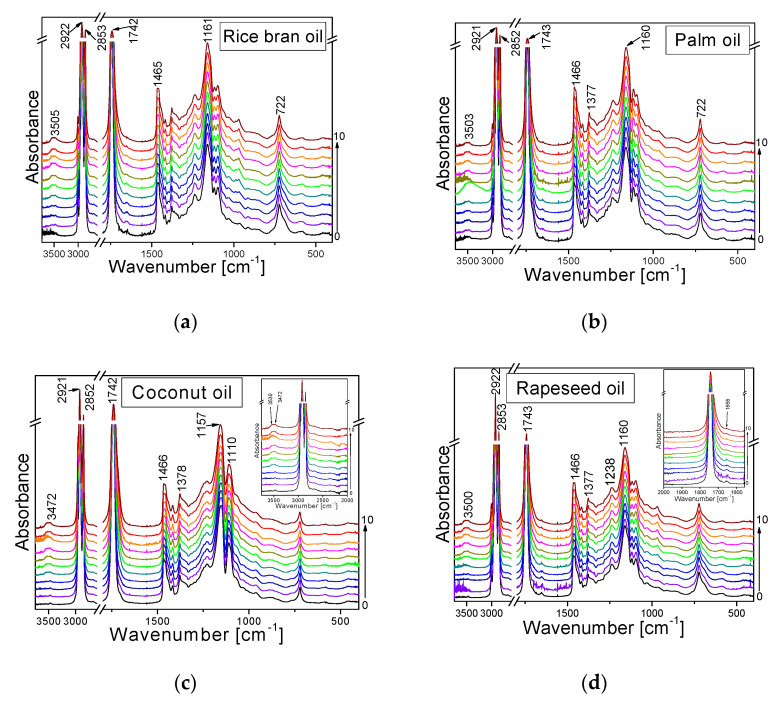
FT-IR spectra of the studied oils: rice bran (**a**), palm (**b**), coconut (**c**), and rapeseed oil (**d**), heated at 180 °C in 10 cycles per 4 h. The spectrum “0” (black color) is for the unheated sample. The spectra are ordered from bottom to top according to the increasing number of cycles.

Figure 2 depicts the FT-IR (Fourier-transform infrared spectroscopy) spectra of the investigated oils. For each oil, the spectra of ten samples were registered for successive heating cycles.

The reactions taking place during the long-term heating of oils were theoretically recognized, defined, and described in the literature. The fatty acids undergo degradation changes depending on the level of double bonds present in the oil. At oxidative conditions, fatty acids generate several degradation products, such as aldehydes, ketones, epoxides, hydroxy compounds, and others. A conjugated double-bond system and *trans*-fatty acids can also be formed. Chemical changes consistent with the degradation of the C=C bond in oxidation and the formation of new toxic substances were identified in the Raman and IR spectra of other oils. They should be visible in the IR spectra presented in Figure 2. The approximate wavenumbers of the following bands are observed in the IR spectra of all oils: 3472—ν(OH); 3005—ν_as_(=C-H)_trans_; 2921—ν_as_(CH_3_/CH_2_); 2852—ν_s_(CH_3_/CH_2_); 1742—ν(C=O); 1658—ν(C=C)_cis_; 1640—ν(C=C)_trans_; 1457, 1436—δ_as_(CH_3_/CH_2_); 1378—δ_s_(CH_3_/CH_2_); 1305—τ(CH_2_); 1267—ρ(=C-H); 1157—ν(C-C)_carotene_/ν(C-O); 1110—ν(C-C); 722—γ(C-C-C). Several symptoms of oil degradation are manifested in the spectra. The heating-type changes have been presented in Figure 2 corresponding to the IR spectra of the studied oils.

**Figure 3:**
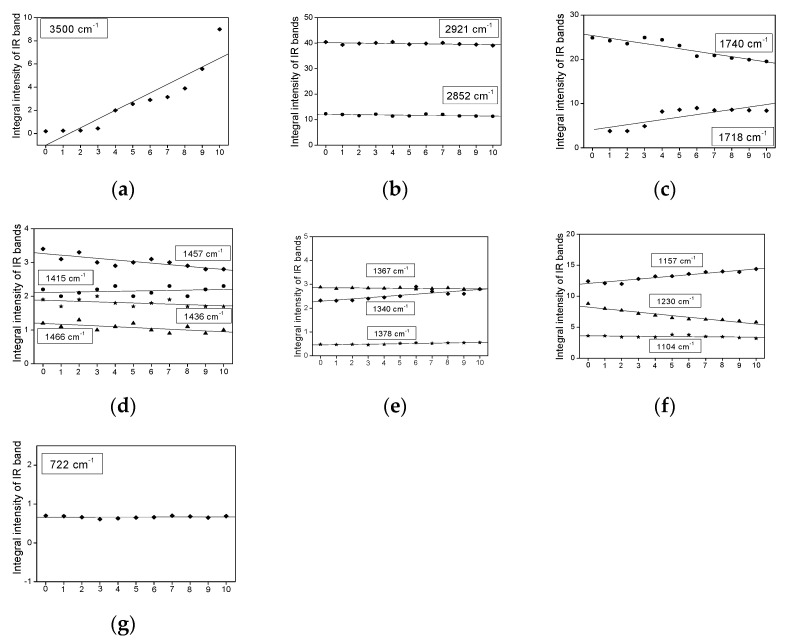
Heating-time dependence of the band intensity observed in the IR spectra of coconut oil at: (**a**) 3500, (**b**) 2921 and 2852, (**c**) 1740 and 1718, (**d**) 1466, 1457, 1436 and 1415, (**e**) 1378, 1367 and 1340, (**f**) 1230, 1157 and 1104, and (**g**) 722 cm^−1^.

**Figure 4:**
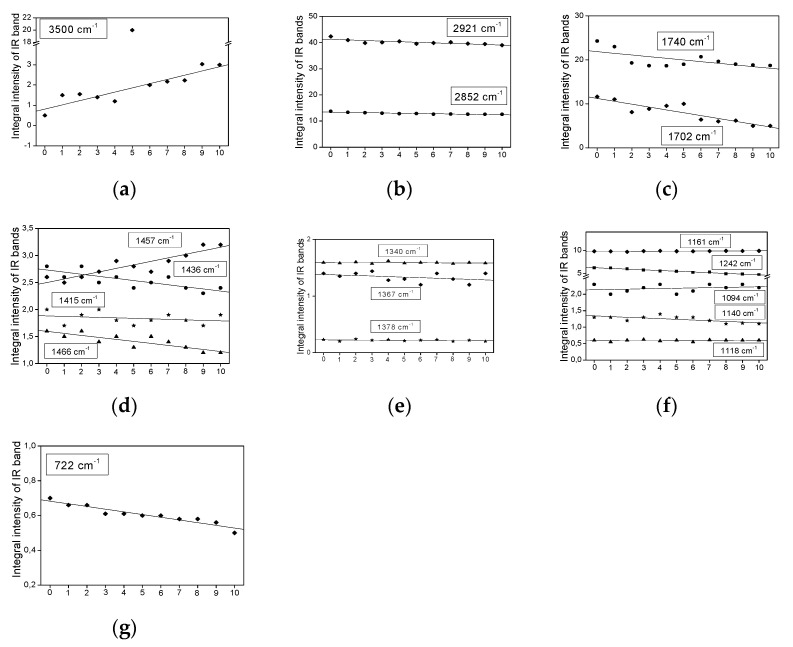
Heating-time dependence of the band intensity observed in the IR spectra of palm oil at: (**a**) 3500, (**b**) 2921 and 2852, (**c**) 1740 and 1702, (**d**) 1466, 1457, 1436 and 1415, (**e**) 1378, 1367 and 1340, (**f**) 1242, 1161, 1140, 1118 and 1094, and (**g**) 722 cm^−1^.

**Figure 5:**
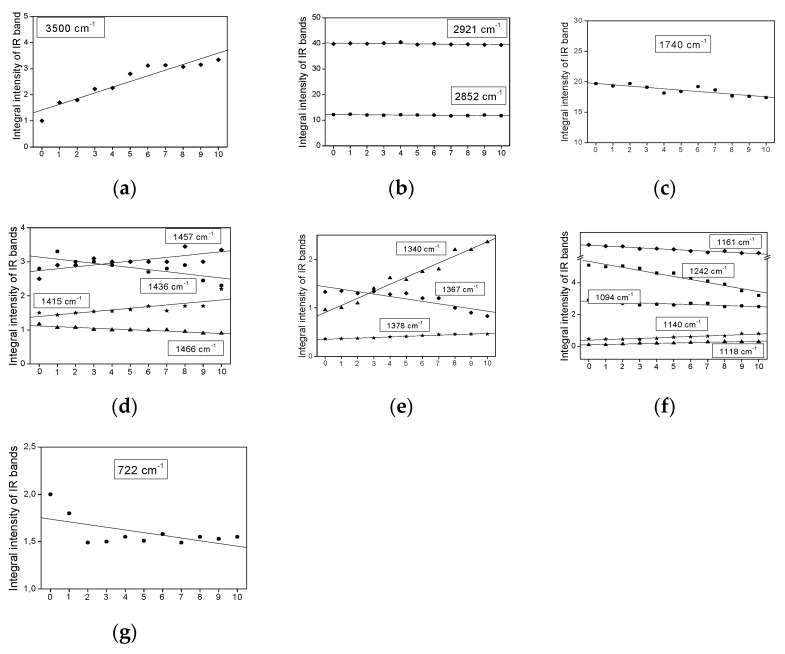
The dependence of the band intensity on heating time, as observed in the IR spectra of the rice bran oil at: (**a**) 3500, (**b**) 2921 and 2852, (**c**) 1740, (**d**) 1466, 1457, 1436 and 1415, (**e**) 1378, 1367 and 1340, (**f**) 1242, 1161, 1140, 1118 and 1094, and (**g**) 722 cm^−1^.

**Figure 6:**
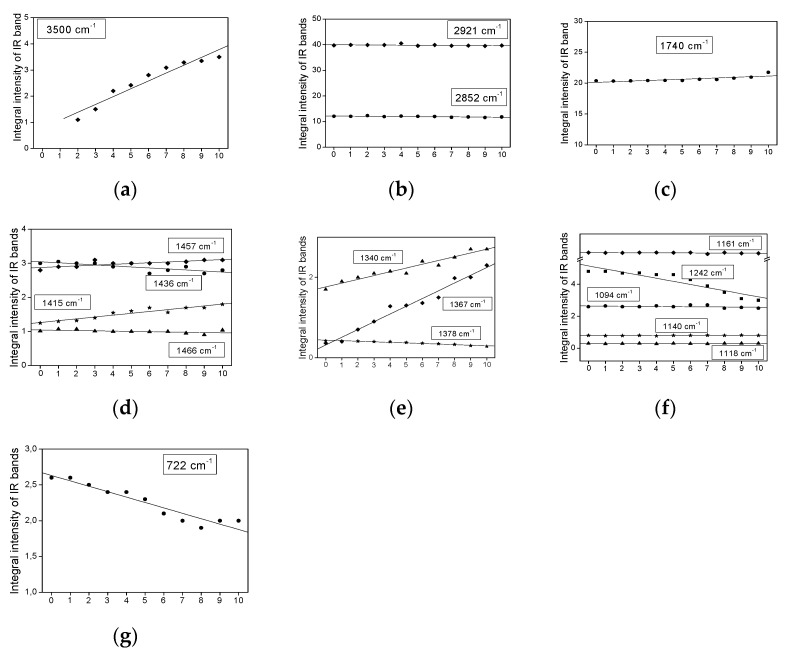
The dependence of the band intensity on heating time, as observed in the IR spectra of the rapeseed oil at: (**a**) 3500, (**b**) 2921 and 2852, (**c**) 1740, (**d**) 1466, 1457, 1436 and 1415, (**e**) 1378, 1367 and 1340, (**f**) 1242, 1161, 1140, 1118 and 1094, and (**g**) 722 cm^−1^.

Figure 3, Figure 4, Figure 5 and Figure 6 show the dependence of the IR bands’ intensity on heating time, observed for the coconut, palm, rice bran, and rapeseed oils, respectively. This order corresponds to the reduction of the unsaturated fraction in these oils.

All the relationships presented in Figure 3, Figure 4, Figure 5 and Figure 6, from the statistical point of view, should be considered linear dependencies because their correlation coefficients are placed in the range of 0.800–0.970. Most of them show a similar direction of change, whether positive or negative. However, some of them exhibit a positive variation in one case and a negative one in the other. Such a situation appears for the bands at 1740, 1718, as well as 1457, 1436, and 1357 cm^−1^, observed mainly for the coconut and rapeseed (canola) oils. They correspond to the following vibrations: ν(C=O), δ_as_(CH_3_) + δ_as_(CH_2_), ρ(=CH) and δ_s_(CH_3_) [29]. This effect probably originates from the fact that these oils are characterized by low smoke points (177 °C for coconut and 107 °C for rapeseed (canola)) and their decomposition appears at a lower temperature than those in the other studied oils.

Three types of thermal dependencies are observed for the bands in the IR spectra of the studied oil samples:

- *The bands do not change their position and intensity during the periodical heating of the oils.*

Such behavior is typical for the majority of the studied oils. This behavior is observed for the bands corresponding to the vibrations of the CH_3_ and CH_2_ groups, i.e., ν_as_(CH_3_/CH_2_)—2981; ν_s_(CH_3_/CH_2_)—2852 (Figure 3b, Figure 4b, Figure 5b and Figure 6b); δ_as_(CH_3_/CH_2_)—1466, 1457, 1436, 1415 (Figure 3d, Figure 4d, Figure 5d and Figure 6d) and δ_s_(CH_3_/CH_2_)—1378, 1367, 1340 (Figure 3e, Figure 4e, Figure 5e and Figure 6e). The methyl and methylene groups are the most stable fragments of the triacyl- and diacyl-glycerol molecules.

- *The bands show an increase in their intensity with increasing heating-time of the oils.*

Such behavior is observed for the ν(OH) stretching vibrations of the hydroxyl group observed at 3500 cm^−1^ (Figure 3a, Figure 4a, Figure 5a and Figure 6a). The increase in the OH groups’ amount in the fatty acids agrees with the thermally induced process >C=C< → >C(OH)—(OH)C< occurring on unsaturated bonds of UFAs acids. This process accompanies the reduction in the *cis* C=C bond amount, which can be seen in the clear decrease in intensity of the ν(C=C)_cis_ band, observed at 1654 cm^−1^. It should be noted that this band is not observed for coconut oil, in which the MUFA + PUFA fraction appears only at 7–8% [28]. The regression coefficients for the band at 3500 cm^−1^ are from 0.75 (coconut oil) to 0.21 (palm oil).

Some characteristic IR bands of the studied oils exhibit a small increase in their intensity (Figure 3c, Figure 4c, Figure 5c, and Figure 6c). Such a time-related heat dependence is shown by the bands observed at 1718 cm^−1^ for coconut oil (Figure 3c; the regression coefficient is 0.57) and at 1740 cm^−1^ for rapeseed oil (Figure 6c; the regression coefficient is 0.10). They correspond to the stretching ν(C=O) vibrations of the ester-carbonyl chromophore. Such changes suggest a small increase in their amount with an increase in the heat-time, which may originate from the decomposition of the ester bonds of acylglycerols and oxidation of the >C=C< groups to >C(OH)-(O)C< bonds.

Similar time-dependent effects are observed for the bands at 1340 and 1157 cm^−1^ for the coconut sample (Figure 3f), at 1457 cm^−1^ for palm oil (Figure 4d), at 1457, 1415, and 1340 cm^−1^ for rice bran oil (Figure 5e), and 1367 and 1340 cm^−1^ for the rapeseed oil (Figure 6e). These changes may suggest a small increase in the CH_2_ groups as a result of the decomposition of the >C=C< bonds and formation of the >CH-HC< units.

- *The bands show a decrease in their intensity with increasing heating time.*

**Figure 7:**
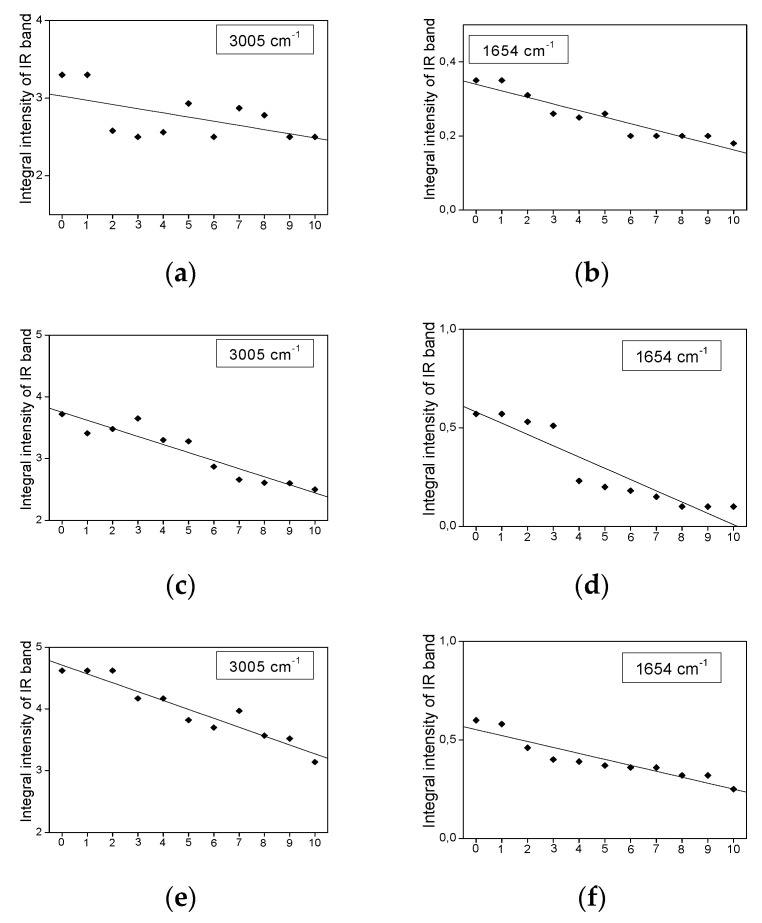
The dependence of the band intensity at 3005 and 1654 cm^−1^ vs. heating time, observed in the IR spectra of palm (**a**,**b**), rice bran (**c**,**d**), and rapeseed (**e**,**f**) oils.

Several bands of the studied IR spectra show a small decrease in their intensity caused by prolonged heating time. They are observed at 1740, 1457, and 1230 cm^−1^ for coconut oil (Figure 3c,d,f), at 3005, 1740, 1702, and 722 cm^−1^ for palm oil (Figure 4c,g and Figure 7a), at 3005, 1740, 1367, 1242, 722, and 691 cm^−1^ for rice bran oil (Figure 5c,e,g and Figure 7c) and at 3005, 1242, 722, and 691 cm^−1^ for rapeseed oil (Figure 6f,g and Figure 7e). These effects are consistent with the structural changes of the acylglycerol molecules. The decrease in the 3005 and 1243 cm^−1^ band intensities confirms the reduction of the amount of =C-H bonds due to the degradation of the C=C double bonds into saturated C-C systems. They originate from the stretching ν(=C-H) and rocking ρ(=C-H) vibrations. The remaining trends originate from the degradation of the small amount of C=O ester-carbonyl (reduction of the C=O bond to the C-OH unit). The decrease in the 692 cm^−1^ band intensities confirms the reduction in the number of C-C-C bonds due to the cracking of the carbon acylglycerol chain.

The IR band appearing at 1654 cm^−1^ in the spectra of palm, rice bran, and rapeseed oils (Figure 7b,d,f) exhibits a clear heating-time dependence. It was assigned to the vibrations of the C=C bond of unsaturated fatty acids. This problem was discussed above as originating from the loss of their unsaturated character.

Figure 7 shows the dependence of the IR band intensities on heating time, observed for the bands at 3005 and 1654 cm^−1^ for the palm, rice bran, and rapeseed oils, respectively. These bands show a decrease in their intensity with the increasing heating time. The regression coefficients for the band at 3005 cm^−1^ are from −0.14 (rapeseed oil) to −0.05 (palm oil), and for the band at 1654 cm^−1^: from −0.06 (rice bran oil) to −0.02 (palm oil).

The bands at 3005 cm^−1^ show a small decrease in their intensity with the increasing heating time. The bands at 1654 cm^−1^ show a significant decrease in their intensity with the increasing heating time.

**Figure 8:**
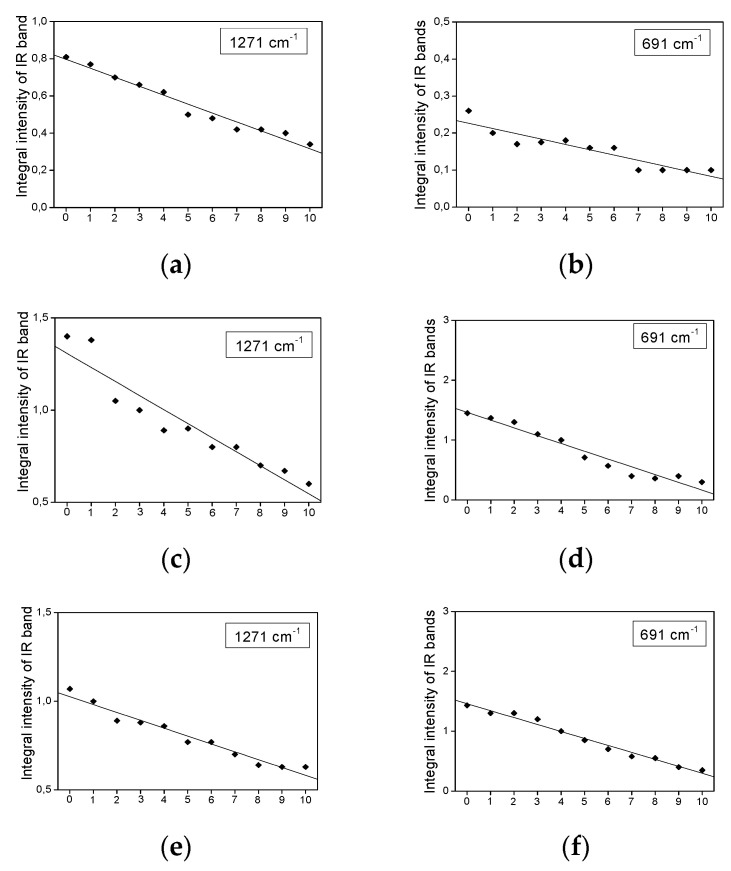
The dependence of the band intensity at 1271 and 691 cm^−1^ vs. heating-time, observed in the IR spectra of palm (**a**,**b**), rice bran (**c**,**d**), and rapeseed (**e**,**f**) oils. The regression coefficients for the band at 1271 cm^−1^ are from −0.08 (rice bran oil) to −0.05 (rapeseed oil) and, for the band at 691 cm^−1^, from −0.13 (rice bran oil) to −0.02 (palm oil).

The palm, rice bran, and rapeseed oils also contain proteins [30,31,32]. It is only coconut oil that does not have these components [33]. The characteristic bands for protein are amides I–IV [34]. The component at 1699 cm^−1^ corresponds to the amide I band (C=O stretching). The component with the wavenumber 1474 cm^−1^ can be attributed to the bending δ(NH_3_^+^), δ(NH_2_), or δ(NH) vibrations of amino groups or amide bonds, probably originating in peptides (amide II band). The Lorentzian components observed at the wavenumber of 1271 cm^−1^ correspond to the vibrations of the amide group (amide III vibration: CN stretching + NH bending); 691 cm^−1^ (amide IV: OCN bending) [34]. The spectra of coconut oil do not contain any component of the amide I–IV bands. There are no changes in the integral intensities for the amide I band, which suggests that the C=O bond is not broken during heating. Integral intensities for amide bands II–IV slightly decrease with heating. The dependence on the intensity of the band at 1474 cm^−1^ (amide II) confirms the change in the content of the proteins in the studied oils (Figure 8).

## 3. Materials and Methods

The commercially available oils used in the studies were purchased from Oleofarm (Mokronoska 8, Wrocław, Poland). Four edible oils were studied: rapeseed (canola), rice bran, coconut, and palm. The oils were heated at 180 °C for 4 h. The heating was repeated in 10 cycles with 24 h intermission between the cycles. The oil samples were collected after each cycle. A total of 44 samples were collected: the unheated sample and samples after each cycle for the four different oils.

The method described in ISO 3961 [35] was applied to determine the iodine values (IV). After dissolving a weighed amount of oil in a mixture of cyclohexane-acetic acid, we added the Wijs solution and stored it for one hour in darkness. A KI-saturated solution was added after the addition of distilled water, and the mix was titrated with sodium phosphate in the presence of starch until the color changed from crimson-purple to bright yellow. The experimental iodine values obtained by the chemical method were compared with those obtained by applying the IR method described in our previous work [29].

FT-IR/ATR spectra were recorded in the range 4000–300 cm^−1^ using a Nicolet 6700 spectrometer (Thermo Fisher Scientific, Waltham, MA, USA) with a portable ATR assembly. The resolution of these measurements was 2 cm^−1^. Each sample was measured in three replicates. The iodine values were obtained from the linear equation I_ν(C=C)_/I_ν(CH2)_ = 7.449 × 10^−4^ × IV − 0.0339, as reported in our earlier work [29].

All evaluations of the iodine values from the spectroscopic data and analysis of the spectral contours were performed using the computer program, ORIGINE 7.5. This analysis included background subtraction and the deconvolution of the experimental bands into Lorentz components. To eliminate an accidental intensity variation in the general intensity level, all the observed bands’ integral intensities were standardized using the statistical R^2^ coefficient. For this purpose, several simulations of the Lorentz deconvolution were performed using a wide and variable number of components. The best-fitting value between the experimental and theoretical spectral course was reached when their statistical R^2^ values were the closest to 1.0.

## 4. Conclusions

In this study, the heating behaviors of coconut, palm, rice bran, and rapeseed oils have been studied. The oils were subjected to thermal decomposition at 180 °C under controlled heating in 10 cycles. One cycle of effective frying lasted 4 h. No other food products or chemicals were added to the oils during the process. Samples were taken after each heating cycle and their iodine values were determined, to verify the effect of prolonged high-temperature treatment on the structure of the tested oils. The iodine values of the heated oils, determined from the IR spectra measurements, show a significant decrease in their degree of unsaturation level. Each sample of the heating cycle was also examined by infrared spectroscopy.

The long-term heating of the oils causes clear changes to the IR spectral contours in the ranges 3600–3400, 1700–1600, and 1000–900 cm^−1^, corresponding to the ν(OH), ν(C=C), and γ(OH) vibrations. These changes prove that in the deep-heating of the oils, the decomposition of the plant fat into fatty acids appears together with the reduction of certain bonds (e.g., C=C, =C-H, and C=O) and the cracking of the acylglycerol chains. This work introduces a new approach to the investigation of the repetitive thermal degradation of edible oils using infrared spectroscopy, the results of which are independently verified by iodine values.

The influence of heating time on the band intensity of proteins was also studied.

During the deep heating of the oils, the decomposition of the plant fats into fatty acids appears together with the reduction of certain bonds and the cracking of the acylglycerol chains. The results show that the C=C bonds are reduced. This is evidenced by the significant reduction in the intensity of the bands, characteristic of ν(C=C) vibrations, and by a slight reduction—ν(=C-H), ρ(=C-H). When some bonds are broken, others arise, e.g., > C(OH)—(HO)C < bonds; > C(OH)—(O)C< bonds; >CH—HC< bonds.

With the increasing heating time, fatty acid chains are broken. This is evidenced by reducing the integral intensities of the bands characteristic of γ(C-C-C) vibration (Figure 3g, Figure 4g, Figure 5g, and Figure 6g). With the cracking of the carbon chain, fewer CH_2_ groups cannot be found as new CH_3_ groups are formed. The bands characteristic of the vibrations of the CH_2_ and CH_3_ groups are in the same position. Therefore, changes in the integral intensity of bands characteristic of vibrations in these groups according to the heating time of the oils are not noticeable. All these relationships between the chemical properties of the studied oils are reflected in the measurements of their IR spectra.

## Figures and Tables

**Table 1:** Amounts of fatty acids in the studied oils.

Oils	SFA	MUFA	PUFA
Palm	46.10	44.73	9.17
Coconut	94.60	4.55	0.85
Rice bran	16.62	45.54	37.84
Rapeseed	8.91	63.08	28.07

## Data Availability

The data presented in this study are available on request from the corresponding author.
